# Development and validation of genome-wide InDel markers with high levels of polymorphism in bitter gourd (*Momordica charantia*)

**DOI:** 10.1186/s12864-021-07499-0

**Published:** 2021-03-16

**Authors:** Junjie Cui, Jiazhu Peng, Jiaowen Cheng, Kailin Hu

**Affiliations:** 1 Department of Horticulture, Foshan University, Foshan, Guangdong 528225 People’s Republic of China; 2 Guangzhou Academy of Agricultural Sciences, Guangzhou, Guangdong 510335 People’s Republic of China; 3 College of Horticulture, South China Agricultural University, Guangzhou, Guangdong 510642 People’s Republic of China

**Keywords:** Bitter gourd, Insertion and deletion (InDel), Molecular marker, Polymorphism, Genetic map

## Abstract

**Background:**

The preferred choice for molecular marker development is identifying existing variation in populations through DNA sequencing. With the genome resources currently available for bitter gourd (*Momordica charantia*), it is now possible to detect genome-wide insertion-deletion (InDel) polymorphisms among bitter gourd populations, which guides the efficient development of InDel markers.

**Results:**

Here, using bioinformatics technology, we detected 389,487 InDels from 61 Chinese bitter gourd accessions with an average density of approximately 1298 InDels/Mb. Then we developed a total of 2502 unique InDel primer pairs with a polymorphism information content (PIC) ≥0.6 distributed across the whole genome. Amplification of InDels in two bitter gourd lines ‘47–2–1-1-3’ and ‘04–17,’ indicated that the InDel markers were reliable and accurate. To highlight their utilization, the InDel markers were employed to construct a genetic map using 113 ‘47–2–1-1-3’ × ‘04–17’ F_2_ individuals. This InDel genetic map of bitter gourd consisted of 164 new InDel markers distributed on 15 linkage groups with a coverage of approximately half of the genome.

**Conclusions:**

This is the first report on the development of genome-wide InDel markers for bitter gourd. The validation of the amplification and genetic map construction suggests that these unique InDel markers may enhance the efficiency of genetic studies and marker-assisted selection for bitter gourd.

**Supplementary Information:**

The online version contains supplementary material available at 10.1186/s12864-021-07499-0.

## Background

DNA-based molecular markers have been available for more than 30 years and are important for plant breeding via molecular marker-assisted selection (MAS) [1–3]. The key breakthrough of DNA-based molecular markers was driven by the invention of polymerase chain reaction (PCR) technology [4]. PCR-based markers have progressively boarded the stage of genetic research such as genetic mapping and gene tagging. Of the PCR-based molecular markers, simple sequence repeat (SSR) and insertion and deletion (InDel) polymorphisms have become the most representative and commonly used markers because they are highly reliable, simple to use, co-dominant, and relatively abundant [1, 5, 6].

A substantial amount of genetic variation is caused by InDels, which is second only to single nucleotide polymorphisms (SNPs), whereas an order of magnitude higher than SSRs [5, 7, 8]. InDel markers combine the characteristics of both SSR and SNP markers, in particular integrating advantages of abundance and simplicity. Thus, InDel markers are a valuable complement for both SSR and SNP markers in genetic studies [9, 10]. The development of InDel markers is becoming readily accessible because of the rapid development of next-generation sequencing (NGS). In crop species such as rice, maize, and soybean, genome-wide InDel markers have been developed based on sequencing data from two accessions [8, 11–13] and among diverse populations [14, 15]. The latter cases certainly can provide more comprehensive and informative InDel markers for the species.

Bitter gourd (*Momordica charantia*), also known as bitter melon, bitter cucumber, and African cucumber, is an important vegetable crop widely distributed and cultivated throughout the tropics [16]. Bitter gourd fruits have many culinary uses in different countries, for example, in China, they are often stir-fried with eggs, meats, and other vegetables, stuffed (stuffed bitter gourd), or added in soups; in India, they are often served with yogurt, mixed with curry, or stuffed with spices and then fried in oil [17]. In addition, bitter gourd has been used in various herbal medicine systems and is associated with a wide range of beneficial effects on health such as anti-diabetic [18–20], anti-HIV [21, 22], and anti-tumor [23, 24]. Like most crops, genetic improvement of bitter gourd is also the challenge faced by breeders, thus developing efficient breeding protocols using molecular markers is required.

Genome-wide SSRs markers have been developed for bitter gourd based on the recently published whole genome sequence [25–27]; however, no work has been done on InDel identification and marker development to date. In this study, using the Dali-11 genome as a reference, we identified the genome-wide InDels from resequencing data of 61 Chinese bitter gourd accessions [27]. Based on the polymorphic information content (PIC), we selected and designed a set of highly informative, unique InDel markers. Moreover, using the newly developed InDel markers, we validated their amplification in two bitter gourd inbred lines, ‘47–2–1-1-3’ and ‘04–17,’ and constructed an InDel genetic map by genotyping the F_2_ population derived from a cross between ‘47–2–1-1-3’ and ‘04–17.’ The results from this study provide a valuable marker resource for bitter gourd research and application in MAS.

## Results

### Identification and distribution of genome-wide InDels

**Fig. 1:**
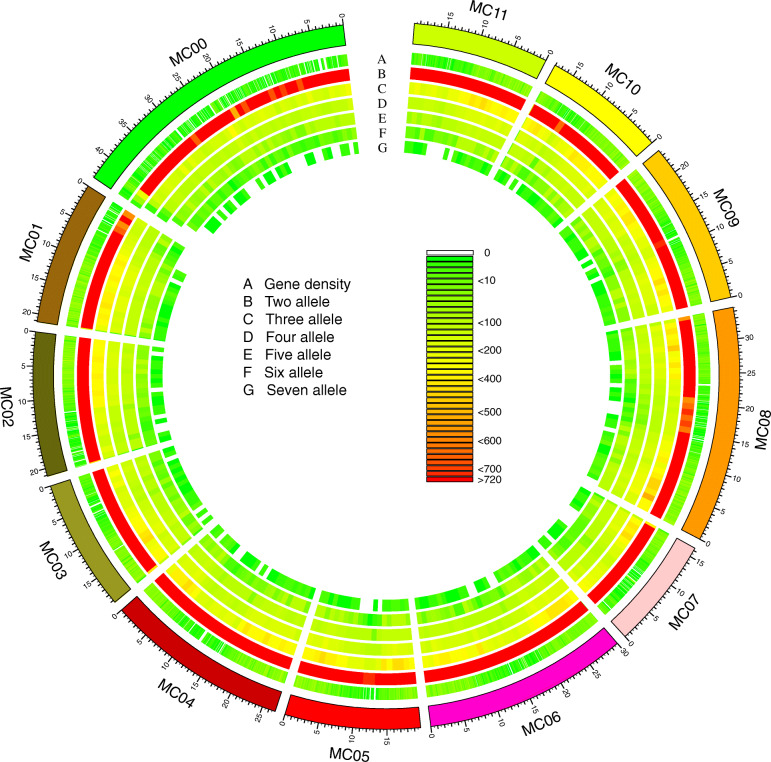
Genome-wide distribution of InDels among the 61 Chinese bitter gourd accessions. Track A denotes the gene density; tracks B to G show the two, three, four, five, six, and seven allele sites, respectively. The unassembled scaffolds or contigs were assigned to MC00 and the data of gene density was cited from a previous report [27]

**Fig. 2:**
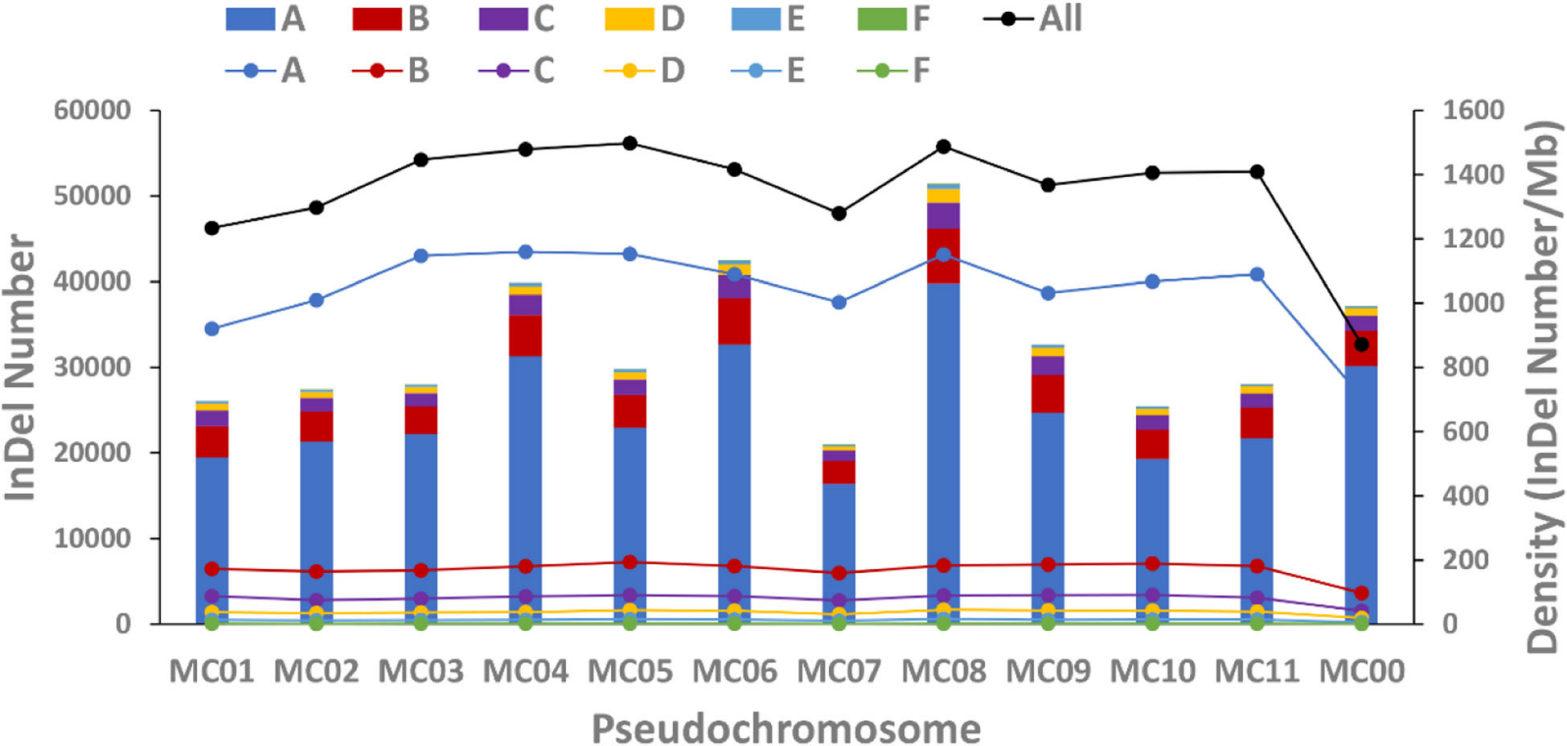
Number and density of InDels identified among 61 Chinese bitter gourd accessions. Bars represent the numbers of InDels; lines represent the density of InDels. A to F indicate the two, three, four, five, six, and seven allele sites; “All” indicates the total number of InDels

In total, 389,487 InDels were identified among the 61 Chinese bitter gourd accessions with an average density of approximately 1298 InDels/Mb across the whole genome (~ 300 Mb). InDels generally are distributed extensively across all 11 pseudochromosomes (MC01-MC11) and in accordance with the distribution of genes (Fig. 1). Polymorphic alleles of InDels were identified in the 61 Chinese bitter gourd accessions, with the number of alleles per InDel ranging from two to seven (Fig. 1; Additional file 1: Table S1). Of these, InDels with two alleles accounted for 77.53% of all InDels, thus were overrepresented. The number of InDels on each pseudochromosome varied from 16,384,005 (MC07) to 34,592,942 (MC08), with the density ranging from 1233 InDels/Mb (MC01) to 1498 InDels/Mb (MC05) (Fig. 2).

### Development of highly polymorphic and unique InDel primers

**Fig. 3:**
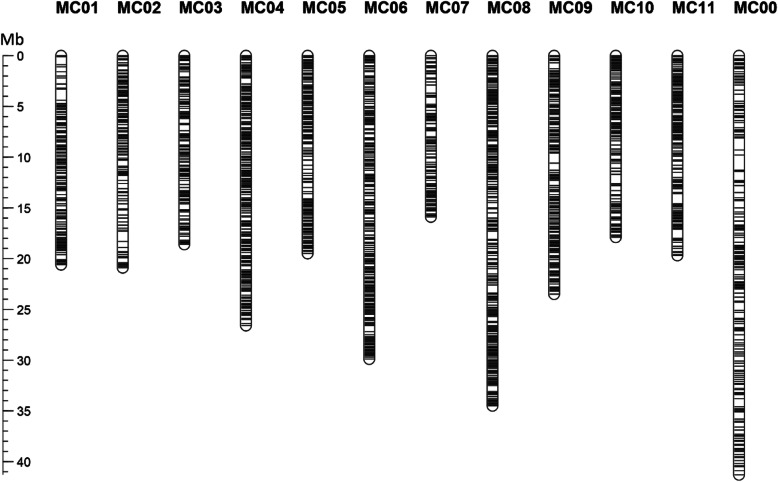
The physical distribution of 2502 unique InDels in the bitter gourd genome

To provide a set of InDels with a high potential for utilization for bitter gourd researchers, we selected 3511 highly polymorphic InDels (MC_g61ind0001–MC_g61ind3511) with PIC ≥0.6 from the 389,487 InDels (Additional file 1: Table S2). Using their flanking sequences retrieved from the ‘Dali-11’ reference genome, a total of 3140 InDel primer pairs were successfully designed by the criteria defined. We subsequently mapped these primer sequences back to the ‘Dali-11’ reference genome and obtained a set of 2502 (79.68%) unique InDel primer pairs (Additional file 1: Table S3), which are distributed throughout the genome (Fig. 3). Then, we evaluated the amplification of the 2502 InDels in two bitter gourd inbred lines, ‘47–2–1-1-3’ and ‘04–17,’ and found that 2466 (98.56%) were successfully amplified. In this study, 212 (8.47%) out of 2502 InDel markers were confirmed to be polymorphic between the two lines (Additional file 2: Figure S1).

### Construction of the InDel genetic map

**Fig. 4:**
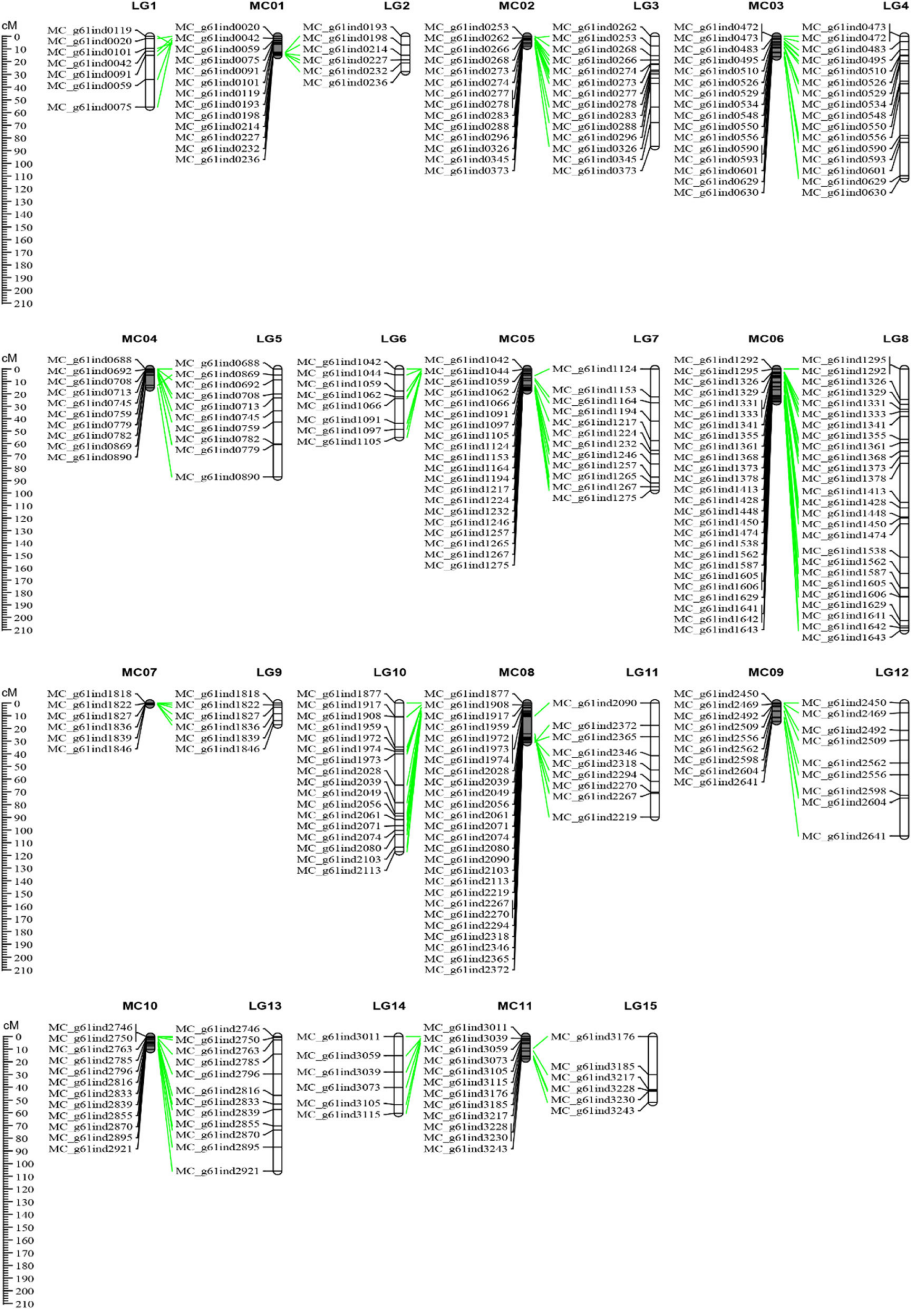
The InDel genetic map of bitter gourd and a comparison with the physical map

In this study, a total of 113 F_2_ individuals derived from the cross between ‘47–2–1-1-3’ and ‘04–17’ were genotyped using the 212 polymorphic InDel markers (Additional file 2: Figure S2). After filtering out 23 markers with severely missing data, 189 InDel markers were loaded into JoinMap 4.0. Finally, a total of 164 markers were integrated into 15 linkage groups (LG; LG1–LG15) (Fig. 4). The total genetic length of the InDel map is 1279.68 cM with an average distance of 7.80 cM between adjacent markers, and the genetic length for each LG ranged from 17.07 (LG9) to 210.70 cM (LG8) (Table 1). Using the reference genome, the InDels on each of the 15 LGs could be assigned to a location and compared with the corresponding 11 pseudochromosomes (MC01–MC11). The genetic and physical position of the InDels on the LGs and the psudochromosomes were highly consistent (Fig. 4). The physical coverage by this map is 148.06 Mb (Table 1), which accounted for approximately half of the ‘Dali-11’ reference genome (~ 300 Mb). Based on the genetic and physical distance, the overall recombination rate of bitter gourd was calculated to be 8.64 cM/Mb.

**Table 1:** Summary of the InDel genetic map of bitter gourd

Linkage group	Pseudochromosome	Marker No.	Genetic distance (cM)	Marker density (cM)	Physical distance (Mb)	Recombination rate (cM/Mb)
LG1	MC01	7	55.64	7.94	6.30	8.83
LG2	MC01	6	27.84	4.64	2.49	11.18
LG3	MC02	14	86.72	6.19	7.82	11.09
LG4	MC03	16	112.04	7.00	16.14	6.94
LG5	MC04	10	86.93	8.69	15.11	5.75
LG6	MC05	8	55.19	6.90	4.02	13.73
LG7	MC05	12	97.78	8.15	12.19	8.02
LG8	MC06	26	210.70	8.10	26.34	8.00
LG9	MC07	6	17.07	2.85	1.46	11.69
LG10	MC08	17	117.06	6.89	10.72	10.92
LG11	MC08	9	89.78	9.98	6.77	13.26
LG12	MC09	9	104.74	11.64	14.50	7.22
LG13	MC10	12	106.04	8.84	10.22	10.38
LG14	MC11	6	60.38	10.06	5.72	10.56
LG15	MC11	6	51.77	8.63	8.26	6.27
Total		164	1279.68	7.80	148.06	8.64

## Discussion

Bitter gourd is an economically important cucurbit crop. Molecular breeding for bitter gourd is far behind that of other cucurbits, such as cucumber and melon, because there is a lack of useful molecular markers. The two recently published bitter gourd genomes and resequencing data of diverse samples have led to the rapid identification of genome-wide polymorphisms that can be utilized for molecular studies [26, 27]. InDel polymorphism is one of the most widely used PCR-based marker systems in MAS strategy. InDel markers have been extensively used in genetic mapping [13, 28] and gene tagging [29–31].

This study accomplished the first large-scale investigation of genome-wide InDels in the bitter gourd genome, with the overall aim of providing a unique, polymorphic set of primers for molecular breeding research. In the present study, we identified a total of 389,487 InDels, which is twice the number of available SSR sites [25], from 61 Chinese bitter gourd accessions. Therefore, we have provided abundant candidates for InDel marker development. The average density of InDels in bitter gourd was observed to be approximately 1298 InDels/Mb, which is greater than the number of InDel markers available for cucumber (916 InDels/Mb) [32] and pepper (71 InDels/Mb) [33], but lower than that in rice (6245 InDels/Mb) [15] and tomato (1448 InDels/Mb) [34]. Moreover, we found that the identification criteria of each study was unique and the number of InDels obtained was largely dependent on the genetic variation of the genotypes from which they were identified. Because the InDels were identified from 61 diverse accessions of Chinese bitter gourd, these InDels will have utility in genetic research on Chinese bitter gourd germplasm and will potentially be useful for materials from other geographic regions.

In addition to the value of a large number of markers in downstream genetic research, highly polymorphic sites that can be PCR amplified are more valuable for marker development. Highly variable sites will ensure the utility of InDel markers in a wider range of bitter gourd germplasm. Therefore, to determine the highly polymorphic InDels in bitter gourd, we screened 2502 unique InDel markers that had a PIC ≥0.6 from the total of 389,487 InDels. This screening criterion is higher than that of PIC ≥0.5 in maize [14] and rice [15]. The experimental PCR validation of the InDel markers between inbred lines ‘47–2–1-1-3’ and ‘04–17’ showed that 212 (8.47%) of 2502 InDel markers were polymorphic, which is lower than expected. We estimated that the polymorphism of this set of 2502 unique InDel markers would be better verified in more bitter gourd materials.

Some molecular marker systems, such as amplified fragment length polymorphisms (AFLP) [35], SSRs, sequence-related amplified polymorphism (SRAP) [36], and SNPs [26, 37, 38], have been used to construct genetic maps of bitter gourd. To the best of our knowledge, no previously published study has developed InDel markers to construct a genetic map of bitter gourd. In the present study, 164 new InDel markers were mapped into 15 LGs covering approximately half of the genome, and the genetic position on 15 LGs were nearly consistent with the physical position on all 11 pseudochromosomes, supporting the accuracy of the assembly of the ‘Dali-11’ reference genome [27]. The overall recombination rate observed in this study is comparable to that previously estimated by a RAD-based genetic map [38]. Taken together, the high amplification rate, number of polymorphisms, and the genetic mapping of this new set of InDel markers can be used for genetic studies such as mapping of the bitter gourd traits.

## Conclusions

Here we report the first analysis of genome-wide InDels distributed throughout the bitter gourd genome and we developed a set of unique and potentially useful InDel markers. We also experimentally validated the amplification of the InDels in the inbred lines ‘47–2–1-1-3’ and ‘04–17’ to determine the polymorphisms. The polymorphic markers were used to construct the first InDel genetic map based on a ‘47–2–1-1-3’ × ‘04–17’ F_2_ population of bitter gourd. The findings of this study indicate that the InDel makers developed in this study are informative and useful in future bitter gourd genetic studies.

## Methods

### Plant materials and genome sequence resources

The whole genome reference sequence of the cultivated bitter gourd line ‘Dali-11’ (*M. charantia*; available at CNGB Nucleotide Sequence Archive, CNSA) (https://db.cngb.org/cnsa/home/; accession: CNP0000016) was anchored onto 11 pseudochromosomes (MC01 to MC11; unanchored scaffolds or contigs were assigned to MC00) [27]. The genomes of 61 diverse Chinese bitter gourd accessions were re-sequenced and their sequence data have been deposited at CNSA (CNP0000017).

Two bitter gourd inbred lines, ‘47–2–1-1-3’ and ‘04–17,’ were used to validate the amplification of InDel markers. A total of 113 F_2_ individuals obtained from crosses of ‘47–2–1-1-3’ (female parent) and ‘04–17’ (male parent) were used to construct the genetic maps. The two parents and 113 F_2_ individuals were grown in Haikou, China (N 20.05°, E 110.20°) in spring 2014. Fresh leaves of the F_2_ individuals were collected for DNA extraction.

### InDel identification and selection in populations

Paired-end, clean reads of 61 Chinese bitter gourd accessions were mapped on the ‘Dali-11’ reference genome with BWA software [39] and exported as a BAM file. Samtools (http://samtools.sourceforge.net) and Picard (http://broadinstitute.github.io/picard) were used to refine the mapping output of BWA. The GATK pipeline [40] was used to detect InDels for each sample. Small insertions and deletions (≤50 bp in length) were calculated. The allelic diversity of each InDel in 61 bitter gourd samples was assessed by polymorphism information content (PIC), which was defined as $\mathrm{PIC}=1-{\sum}_{i=1}^n{P}_i^2-{\sum}_{i=1}^{n-1}{\sum}_{j=i+1}^n2{P}_i^2{P}_j^2$, where P_i_ and P_j_ is the frequency of the i and j allele, respectively, and n is the allele number. InDel loci with PIC ≥0.6 were retained for primer design.

### Designing unique InDel primers and validation of PCR amplification

BatchPrimer3 (https://wheat.pw.usda.gov/demos/BatchPrimer3/) [41] was used to design InDel primers following the conditions described in a previous study [25]. Specifically, the InDel primers were designed to have the following characters: primer size, 18–27 bp with an optimum length of 20 bp; primer melting temperature (*T*m), 57.0–63.0 °C with an optimum temperature of 60 °C; product size, 100–500 bp with an optimum size of 250 bp; and primer GC content, 40–60% with an optimum GC content of 50%. All the designed primer pairs were anchored back onto the ‘Dali-11’ reference genome. Primer pairs were defined as unique if both the forward and reverse primers were uniquely aligned to the reference genome.

The PCR assay was conducted in a total reaction volume of 20 μL containing 20 ng of genomic DNA, 100 μM dNTPs (Eastwin, Guangzhou, China), 0.1 μM of each forward and reverse primer, 0.5 U Taq DNA polymerase (Eastwin, Guangzhou, China), 2.0 μL of 10 × Taq buffer, and 2.0 mM MgCl_2_. PCR amplification was conducted under the following conditions: initial denaturation of 5 mins at 94 °C; followed by 25 cycles of 30 s at 94 °C, 30 s at 60 °C, and 1 min at 72 °C; and a final extension of 5 mins at 72 °C. Then 2–4 μL of the amplified products were used for electrophoresis, which was run on a 6% polyacrylamide gel.

### Genetic map construction

JoinMap 4.0 software [42] was used to construct the genetic map. The independence logarithm of the odds (LOD) score was set to a threshold range of 3.0 to 10.0. A regression analysis with Kosambi’s function was used to estimate genetic distances. The genetic and physical maps were drawn using MapChart version 2.2 software [43].

## Supplementary Information

**Additional file 1: Table S1.** The number of InDels with varying numbers of alleles. **Table S2.** List of 3511 polymorphic InDels with PIC ≥0.6. **Table S3.** List of 2502 unique InDel primer pairs.

**Additional file 2: Figure S1.** *Indel polymorphisms between* ‘04–17’and ‘47–2–1-1-3′. **Figure S2.** One of the polymorphic marker MC_g61ind2372 amplified in 113 F2 individuals from crosses of ‘04–17′ and ‘47–2–1-1-3′..

## Data Availability

All the genomic sequence information generated in the current study has been deposited at CNGB Nucleotide Sequence Archive, CNSA) (CNP0000016 and CNP0000017).

## Abbreviations

InDels: Insertion deletions
PIC: Polymorphism information content
MAS: Marker-assisted selection
PCR: Polymerase chain reaction
SSR: Simple sequence repeat
NGS: Next-generation sequencing
LG: Linkage group
RFLP: Restriction fragment length polymorphism
SRAP: Sequence-related amplified polymorphism
SNP: Single nucleotide polymorphism

## References

[1] Schlötterer C (2004). The evolution of molecular markers—just a matter of fashion?. Nat Rev Genet.

[2] Collard BC, Mackill DJ (2008). Marker-assisted selection: an approach for precision plant breeding in the twenty-first century. Philos T R Soc B.

[3] Mohan M, Nair S, Bhagwat A, Krishna T, Yano M, Bhatia C, Sasaki T (1997). Genome mapping, molecular markers and marker-assisted selection in crop plants. Mol. Breed..

[4] Saiki RK, Scharf S, Faloona F, Mullis KB, Horn GT, Erlich HA, Arnheim N (1985). Enzymatic amplification of b-globin genomic sequences and restriction site analysis for diagnosis of sickle cell anemia. Science..

[5] Mills RE, Luttig CT, Larkins CE, Beauchamp A, Tsui C, Pittard WS, Devine SE (2006). An initial map of insertion and deletion (INDEL) variation in the human genome. Genome Res.

[6] Powell W, Machray GC, Provan J (1996). Polymorphism revealed by simple sequence repeat. Trends Plant Sci.

[7] Mullaney JM, Mills RE, Pittard WS, Devine SE (2010). Small insertions and deletions (INDELs) in human genomes. Hum Mol Genet.

[8] Lü Y, Cui X, Li R, Huang P, Zong J, Yao D, Li G, Zhang D, Yuan Z (2015). Development of genome-wide insertion/deletion markers in rice based on graphic pipeline platform. J Integr Plant Biol.

[9] Mahmood S, Li Z, Yue X, Wang B, Chen J, Liu K (2016). Development of INDELs markers in oilseed rape (*Brassica napus* L.) using re-sequencing data. Mol. Breeding..

[10] Väli Ü, Brandström M, Johansson M, Ellegren H (2008). Insertion-deletion polymorphisms (indels) as genetic markers in natural populations. BMC Genet.

[11] Shen YJ, Jiang H, Jin JP, Zhang ZB, Xi B, He YY, Wang G, Wang C, Qian L, Li X (2004). Development of genome-wide DNA polymorphism database for map-based cloning of rice genes. Plant Physiol.

[12] Song X, Wei H, Cheng W, Yang S, Zhao Y, Li X, Luo D, Zhang H, Feng X (2015). Development of INDEL markers for genetic mapping based on whole genome resequencing in soybean. G3-genes Genom. Genet..

[13] Li W, Cheng J, Wu Z, Cheng Q, Tan S, Tang X, Cui J, Li Z, Hu K (2015). An InDel-based linkage map of hot pepper (*Capsicum annuum*). Mol Breed.

[14] Liu J, Qu J, Yang C, Tang D, Li J, Lan H, Rong T (2015). Development of genome-wide insertion and deletion markers for maize, based on next-generation sequencing data. BMC Genomics.

[15] Liu J, Li J, Qu J, Yan S (2015). Development of genome-wide insertion and deletion polymorphism markers from next-generation sequencing data in rice. Rice..

[16] Behera TK, Behera S, Bharathi LK, John KJ, Simon PW, Staub JE. Bitter Gourd: botany, horticulture, breeding. Hortic Rev. 2010;37:101–41.

[17] Lim T (2012). Momordica charantia. Edible medicinal and non-medicinal plants.

[18] Tan MJ, Ye JM, Turner N, Hohnen-Behrens C, Ke CQ, Tang CP, Chen T, Weiss HC, Gesing ER, Rowland A (2008). Antidiabetic activities of triterpenoids isolated from bitter melon associated with activation of the AMPK pathway. Chem Biol.

[19] Khanna P, Jain SC, Panagariya A, Dixit VP (1981). Hypoglycemic activity of polypeptide-p from a plant source. J Nat Prod.

[20] Yang B, Li X, Zhang C, Yan S, Wei W, Wang X, Deng X, Qian H, Lin H, Huang W (2015). Design, synthesis and biological evaluation of novel peptide MC2 analogues from *Momordica charantia* as potential anti-diabetic agents. Org Biomol Chem.

[21] Lee-Huang S, Huang P, Nara P, Chen H, Kung H, Huang P, Huang H, Huang P (1990). MAP 30: a new inhibition of HIV-1 infection and replication. FEBS Lett.

[22] Wang YX, Neamati N, Jacob J, Palmer I, Stahl SJ, Kaufman JD, Huang PL, Huang PL, Winslow HE, Pommier Y (1999). Solution structure of anti-HIV-1 and anti-tumor protein MAP 30: structural insights into its multiple functions. Cell..

[23] Akihisa T, Higo N, Tokuda H, Ukiya M, Akazawa H, Tochigi Y, Kimura Y, Suzuki T, Nishino H (2007). Cucurbitane-type triterpenoids from the fruits of Momordica charantia and their cancer chemopreventive effects. J Nat Prod.

[24] Dia VP, Krishnan HB (2016). BG-4, a novel anticancer peptide from bitter gourd (*Momordica charantia*), promotes apoptosis in human colon cancer cells. Sci Rep.

[25] Cui J, Cheng J, Nong D, Peng J, Hu Y, He W, Zhou Q, Dhillon NPS, Hu K (2017). Genome-wide analysis of simple sequence repeats in bitter gourd (*Momordica charantia*). Front Plant Sci.

[26] Urasaki N, Takagi H, Natsume S, Uemura A, Taniai N, Miyagi N, Fukushima M, Suzuki S, Tarora K, Tamaki M (2017). Draft genome sequence of bitter gourd (*Momordica charantia*), a vegetable and medicinal plant in tropical and subtropical regions. DNA Res.

[27] Cui J, Yang Y, Luo S, Wang L, Huang R, Wen Q, Han X, Miao N, Cheng J, Liu Z (2020). Whole-genome sequencing provides insights into the genetic diversity and domestication of bitter gourd (*Momordica* spp.). Hortic Res.

[28] Zhang XF, Sun HH, Xu Y, Chen B, Yu SC, Geng SS, Wang Q. Development of a large number of SSR and InDel markers and construction of a high-density genetic map based on a RIL population of pepper ( *Capsicum annuum* L.). Mol Breeding. 2016;36(7):92.

[29] Ramkumar G, Srinivasarao K, Mohan KM, Sudarshan I, Sivaranjani AKP, Gopalakrishna K, Neeraja CN, Balachandran SM, Sundaram RM, Prasad MS (2011). Development and validation of functional marker targeting an InDel in the major rice blast disease resistance gene *Pi54* (*Pik*^*h*^). Mol Breed..

[30] Chen T, Zhang YD, Zhu Z, Zhao L, Zhao QY, Zhou LH, Yao S, Xin YU, Wang CL (2014). Development of new InDel marker to detect genotypes of Rf-1a conferring fertility restoration of BT-type cytoplasmic male sterility in rice. Rice Sci.

[31] Lv HH, Yang LM, Kang JG, Wang QB, Wang XW, Fang ZY, Liu YM, Zhuang M, Zhang YY, Lin Y (2013). Development of InDel markers linked to Fusarium wilt resistance in cabbage. Mol Breed..

[32] Qi J, Liu X, Shen D, Miao H, Xie B, Li X, Zeng P, Wang S, Shang Y, Gu X (2013). A genomic variation map provides insights into the genetic basis of cucumber domestication and diversity. Nat Genet.

[33] Qin C, Yu C, Shen Y, Fang X, Chen L, Min J, Cheng J, Zhao S, Xu M, Luo Y (2014). Whole-genome sequencing of cultivated and wild peppers provides insights into *Capsicum* domestication and specialization. P Natl Acad Sci USA.

[34] Lin T, Zhu G, Zhang J, Xu X, Yu Q, Zheng Z, Zhang Z, Lun Y, Li S, Wang X (2014). Genomic analyses provide insights into the history of tomato breeding. Nat Genet.

[35] Kole C, Olukolu BA, Kole P, Rao VK, Bajpai A, Backiyarani S, Singh J, Elanchezhian R, Abbott AG (2012). The first genetic map and positions of major fruit trait loci of bitter melon (*Momordica charantia*). J Plant Sci Mol Breed.

[36] Wang ZS, Xiang CP (2013). Genetic mapping of QTLs for horticulture traits in a F2-3 population of bitter gourd (*Momordica charantia* L.). Euphytica..

[37] Matsumura H, Miyagi N, Taniai N, Fukushima M, Tarora K, Shudo A, Urasaki N (2014). Mapping of the gynoecy in bitter gourd (*Momordica charantia*) using RAD-Seq analysis. Plos One.

[38] Cui J, Luo S, Niu Y, Huang R, Wen Q, Su J, Miao N, He W, Dong Z, Cheng J (2018). A RAD-based genetic map for anchoring scaffold sequences and identifying QTLs in bitter gourd (*Momordica charantia*). Front Plant Sci.

[39] Li H, Handsaker B, Wysoker A, Fennell T, Ruan J, Homer N, Marth G, Abecasis G, Durbin R (2009). 1000 genome project data processing subgroup. The sequence alignment/map format and SAMtools. Bioinformatics..

[40] Mckenna A, Hanna M, Banks E, Sivachenko A, Cibulskis K, Kernytsky A, Garimella K, Altshuler D, Gabriel S, Daly M (2010). The genome analysis toolkit: a MapReduce framework for analyzing next-generation DNA sequencing data. Genome Res.

[41] You FM, Huo N, Gu YQ, Luo MC, Ma Y, Hane D, Lazo GR, Dvorak J, Anderson OD (2008). BatchPrimer3: a high throughput web application for PCR and sequencing primer design. BMC Bioinformatics.

[42] Van Ooijen JW. Joinmap 4: software for the calculation of genetic linkage maps in experimental populations. Wageningen: Kyazma BV; 2006.

[43] Voorrips RE (2002). MapChart: software for the graphical presentation of linkage maps and QTLs. J Hered.

