# Supplementary material for: Development and validation of genome-wide InDel markers with high levels of polymorphism in bitter gourd (Momordica charantia)
Source: BMC Genomics. 2021 Mar 16;22:190. doi: 10.1186/s12864-021-07499-0 (PMC7968231; doi:10.1186/s12864-021-07499-0)
Supplement: Supplementary file 2 — Additional file 2: Figure S1. Indel polymorphisms between ‘04–17’and ‘47–2–1-1-3′. Figure S2. One of the polymorphic marker MC_g61ind2372 amplified in 113 F2 individuals from crosses of ‘04–17′ and ‘47–2–1-1-3′.. [file 12864_2021_7499_MOESM2_ESM.docx]

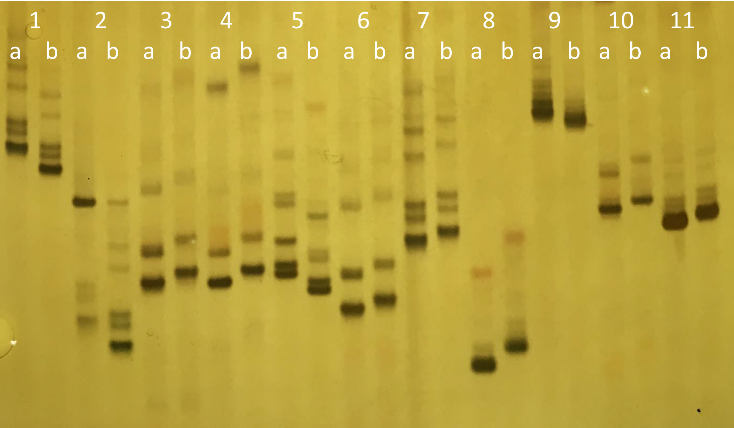


**Fig. S1** InDel polymorphisms between ‘04-17’and ‘47-2-1-1-3’. PCR products from lines 1 to 11 were amplified by MC_g61ind0253, MC_g61ind0256, MC_g61ind0262, MC_g61ind0266, MC_g61ind0268, MC_g61ind0273, MC_g61ind0274, MC_g61ind0277, MC_g61ind0279, MC_g61ind0281, and MC_g61ind0283, respectively. a: inbred line ‘04-17’; b: inbred line ‘47-2-1-1-3’.


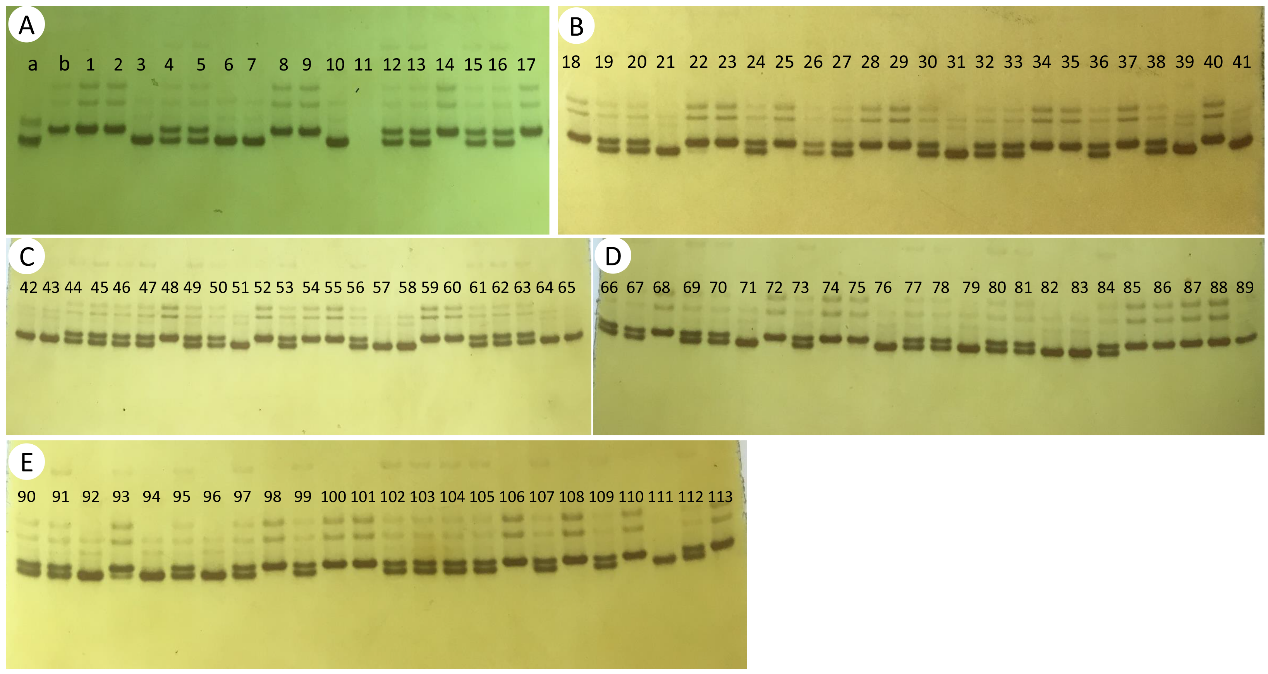


**Fig. S2** One of the polymorphic marker MC_g61ind2372 amplified in 113 F_2_ individuals from crosses of ‘04-17’ and ‘47-2-1-1-3’. a: inbred line ‘04-17’; b: inbred line ‘47-2-1-1-3’.
